# An epidemiological study of cervical and breast screening in India: district-level analysis

**DOI:** 10.1186/s12905-020-01083-6

**Published:** 2020-10-07

**Authors:** Monica, Raman Mishra

**Affiliations:** International Institute for Population Sciences, Mumbai, India

**Keywords:** Cervical screening, Breast screening, Spatial analysis, India

## Abstract

**Background:**

Breast cancer and cervical cancer, the most common forms of cancer in women worldwide, are on a fast and steady rise, accounting for more deaths in women than any other cancer in the developing world. Cancer screening tests are an important tool to combat cancer-related morbidity and mortality. World Health Organization aims to accelerate action to achieve Goal 3.4 of the Sustainable Development Goals (SDG 3.4) in order to reduce premature mortality from non-communicable disease, including cancer by one-third by 2030. This study aims to examine the geospatial variation of cervical and breast screening across districts and to identify factors that contribute to the utilization of screening among women in India.

**Methods:**

Until recently, there was no evidence pertaining to screening for cervical and breast cancers at the national level. Information on examination of the breast and cervix from over 699,000 women aged 15–49 years was collected for the first time in the fourth round of National Family Health Survey, 2015–16 (NFHS-4). For the present study, the data were aggregated for all 640 districts in India. Moran’s Index was calculated to check for spatial autocorrelation. Univariate Local Indicators of Spatial Association (LISA) maps were plotted to look for spatial dependence associated with the uptake of screening practices. The spatial error model was employed to check for spatial magnitude and direction.

**Results:**

The common factors associated with uptake of both cervical and breast screening at the district level were; women belonging to a general caste, residing in rural areas, being currently married, and being well-off economically. Being insured was positively associated with the uptake of cervical screening only. This study provides spatial inference by showing geographical variations in screening of cervix and breast across districts of India.

**Conclusions:**

By showing geographical disparities in screening practices across districts of India, this study highlights the importance of ensuring a region-specific and organ-specific approach towards control and prevention of cancer. The identified factors responsible for the uptake of screening could be a guiding force to decide how and where tailored interventions may be best targeted.

## Background

Cancer is increasingly being recognized as a major cause of mortality and morbidity, with approximately 18.1 million new cases reported in 2018 [1]. The World Health Organization (WHO) projects that the number of global cancer deaths will rise by 45% between 2008 and 2030 [2]. The rising burden of the mortality from cancer is likely to be fivefold greater in the low-income countries compared to established market economies [3]. The economic burden of cancer is significant and rising. In 2010, the total annual economic cost of cancer was estimated at approximately US$ 1.6 trillion [4], thus threatening health budgets at all income levels and causing financial distress for individuals and families.

Breast cancer and cervical cancer, the most common forms of cancer in women worldwide, too are on a fast and steady rise, accounting for more deaths in women than any other cancer in the developing world [1]. Statistics suggest that about 527,624 and 1,671,149 new cases of cervical and breast cancers are added every year. To this, India contributes about 122,844 cervical cancer and 144,937 breast cancer cases every year [5]. India accounts for nearly one-third of the global cervical cancer deaths, with women facing a 1.6% cumulative risk of developing cervical cancer and 1.0% cumulative death risk from cervical cancer. Similarly, the cumulative risk of developing breast cancer is 2.7%, and cumulative death risk is 1.5% [6]. Earlier, cervical cancer was the most common cancer throughout the nation, but now the incidence of breast cancer has surpassed it and is the leading cause of death [7]. A vital observation here is that breast and cervical cancers are curable if diagnosed at an early stage. These cancers are preventable with access to high-quality care, periodic screening tests, and regular follow-up [8].

The World Health Assembly (WHA 70.12), in its agenda for cancer prevention and control in the context of an integrated approach, urges governments and the WHO to accelerate action to achieve Goal 3.4 of the Sustainable Development Goals (SDG 3.4) in order to reduce premature mortality from non-communicable diseases including cancer by one-third by 2030 [8]. The strategies to reduce the high burden of cervical and breast cancers include risk factor intervention, vaccination, screening, and early diagnosis [9]. Effective screening is the first step toward reducing the burden of cervical and breast cancers. Screening is defined as “the systematic application of a test or an inquiry to identify individuals at sufficient risk of a specific disorder to warrant further investigation or direct preventive action among persons who have not sought medical attention on account of symptoms of that disorder [10]. On the other hand, screening uptake refers to the proportion of persons eligible to be screened within a population who have been both invited for screening and have received an adequate screening during a specified period [11]. Experience from the developed world shows that effective population-based screening programmes can easily reduce the incidence of cervical and breast cancers. Mortality rates from cervical and breast cancers can also be reduced by such interventions [12, 13]. Despite the clear and proven benefits of population-based screening programs, screening for cervical and breast cancers in low-income countries, including India, remains a challenge.

Until recently, there was no evidence pertaining to screening for cervical and breast cancers in India. Information on examination of the breast and cervix from over 699,000 women aged 15–49 years was collected for the first time in the fourth round of National Family Health Survey, 2015–16 (NFHS-4) [14]. The availability of such information in NFHS-4 provided us with a great opportunity to analyze the levels and patterns in the screening for cervical and breast cancers in India at the state and district levels.

Some past studies, mostly conducted in developed country settings, have identified several socio-economic, demographic, bio-medical, and residence-related characteristics that are associated with the screening of the cervix and breast. The likelihood of a woman receiving a Pap test, or a clinical breast examination, depends on many aspects such as age, marital status, income level, education, and health status. Women with higher education, higher incomes, and greater insurance coverage are more likely to undergo cervical and breast cancer screening services [15]. Employed females are more inclined to go for screening because of their higher opportunity cost, higher incomes, and ability to afford out-of-pocket expenditure [16]. On the other hand, rural women are less likely than urban women to go for cervical and breast screening [11, 17, 18]. Studies of breast and cervical screening show that women with greater access to health care, such as those with health insurance, are opting to have screening tests [16, 19]. The risk of infection with Human papillomavirus (HPV) and also the risk of cervical cancer depends on the number of sexual partners, age at first intercourse, and sexual behavior of the woman’s male partner [20]. Additional risk indicators for cervical cancer are number of live births, long-term use of oral contraceptives, and cigarette smoking [21]. Risk factors, other than socio-economic and demographic characteristics, accountable for breast cancer are alcohol, obesity, longer use of oral contraceptives, early onset of menstrual periods, etc. [22]. Studies also suggest that health policies and quality of the health care system influence cervical and breast cancer screening behaviors [23, 24].

Even though there has been an increase in the prevalence of cancer among the female population, most of the research on cancers in females is concentrated only on the incidence and mortality rates of cervical and breast cancers. A review of cancer screening-related literature in India reveals that the spatial perspective of cancer screening has not been explored yet. The present study attempts to address some of these research gaps.

## Methods

### Aim and rationale for spatial dependence

This study aims to identify factors affecting uptake of cervical and breast screening and to develop a comprehensive assessment of the geographical distribution of screening to capture the precise hotspots of the screening practices at the district level. It is crucial to note that any aggregation of socioeconomic, demographic or health variables over a geographic space tends to manifest a spatial pattern or spatial clustering. In such a case, spatial autocorrelation creates a problem for statistical testing as the autocorrelated data violates the assumptions of classical statistics, one of them being the independence of the observations [25]. Such regression analyses, which ignore the spatial correlations, lead to incorrect inference of the estimated regression coefficients by narrowing the confidence intervals [26]. This limitation can, however, be overcome using geospatial models [27, 28].

### Data source

This study analyzed data from the fourth round of the National Family Health Survey (NFHS, 2015–16). NFHS was conducted under the supervision of the Ministry of Health & Family Welfare, Government of India and harmonized by the International Institute for Population Sciences (IIPS), Mumbai. A two-stage sampling design was adopted in the survey for both rural and urban areas. In the first stage, villages were selected by using the probability proportional to size scheme (PPS) for the rural areas. At the second stage, households were then chosen from the designated villages by using systematic sampling. For the urban areas, first stage of sampling involved selection of census enumeration blocks (CEBs) by using PPS, followed by selection of households using systematic sampling at the second stage. NFHS-4 collected data in all the 29 states and 6 union territories of India, divided administratively into 640 districts. The survey collected information from all eligible women aged 15–49 years, who were asked questions on a large variety of topics, including background characteristics, family planning, fertility preferences and other health issues (tuberculosis, current morbidity- diabetes, asthma, goiter, heart disease, cancer). Districts are the smallest administrative unit in India. Analysis at this level yield meaningful insights.

### Study population

The self-reported information on medical examination (screening) of cervix and breast by the women aged 15–49 years was used in this study. Precisely, the district-level data for all the states and union territories were aggregated for the present study.

### Study variables

The outcome variables used in the analysis were cervical and breast screening. The data of women undergoing cervical and breast screening in the age group 15–49 years was aggregated at the district level. The study assessed the variations in cervical and breast screening through a set of independent factors.

We assessed the following predictors influencing the uptake of cervical screening: covered by insurance, having multiple sexual partners, consuming tobacco in any form, using oral contraceptives, and having parity greater than three. The determinants influencing the uptake of breast screening included: being obese, using oral contraceptives, consuming tobacco in any form, covered by insurance, and consuming alcohol.

Other common socio-demographic factors included in the analysis were: status of literacy (literate), marital status (currently married), religion (Hindu), caste (general), area of residence (rural), and economic status (rich). All the variables were aggregated from the individual level to the district level.

### Statistical analysis

First, the ordinary least squares (OLS) model was estimated to explore the relationship between cervical and breast screening and their independent variables respectively. Second, tests for spatial dependence were performed. Third, the spatial regression models were employed to study the spatial effect on screening.

### Estimation of spatial association

**Fig. 1:**
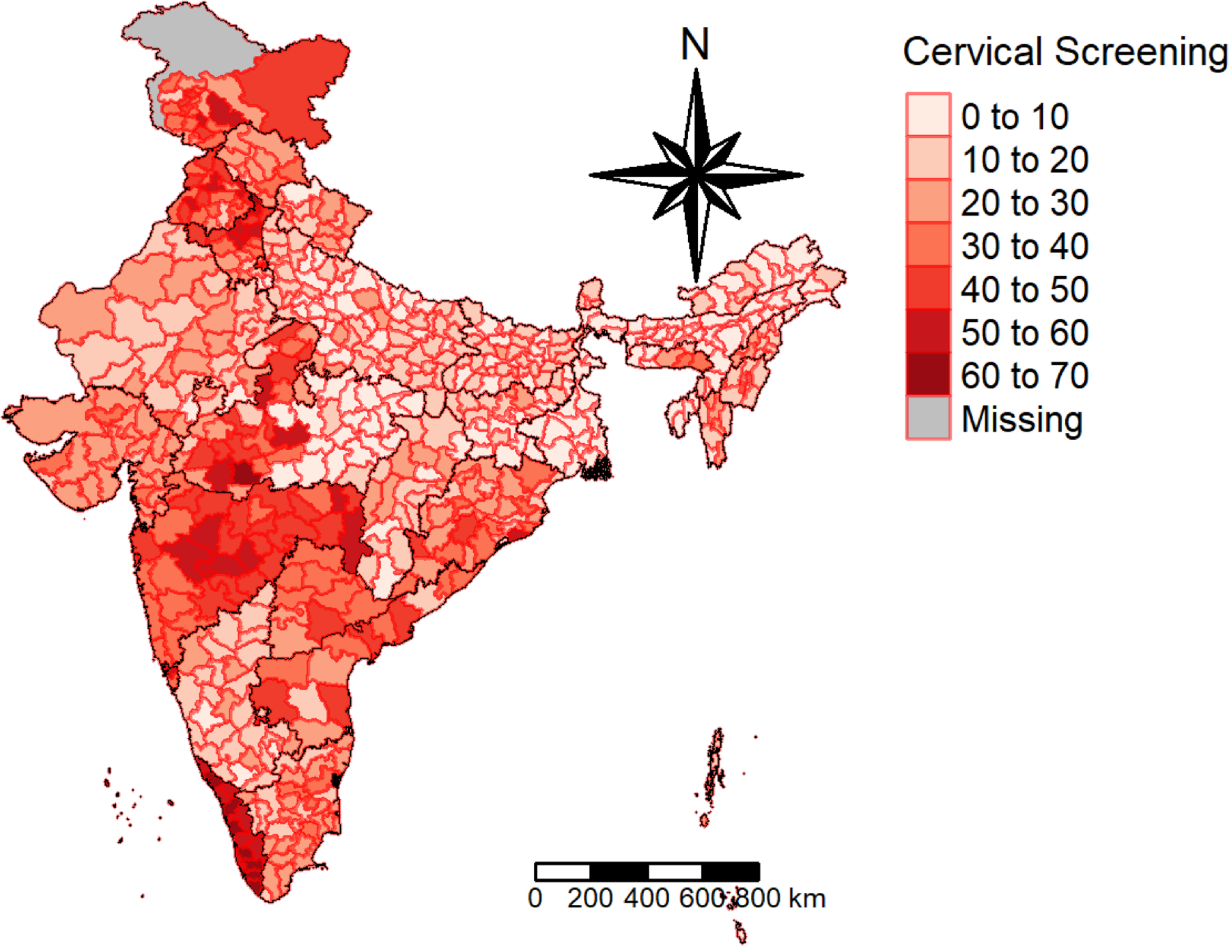
Prevalence of Cervical Screening. The district’s boundaries are as per the Census of India, 2011

We used R software (Version 3.6.1, http://cran.r-project.org/) to generate descriptive maps of cervical and breast screening across the districts of India. We then generated the spatial weights for the calculation of the spatial autocorrelation statistics. Contiguity-based spatial weights were used, as our objective was to understand the spatial interdependence between the dependent variable and a set of independent variables in the neighboring regions (districts). Within the spatially contiguous weights, we chose Queen’s Weight, which works on the principle that at least one point on the boundary of a polygon is within the snap distance of at least a point of its neighbor (Fig. 1 Appendix). Finally, we used geo-spatial techniques, such as Moran’s I statistics, Univariate LISA (Local Indicators of Spatial Association), and geospatial regression, to address the research questions.

### Global and local spatial autocorrelation

Moran’s I is the measure of global spatial autocorrelation. The magnitude of Moran’s I was estimated by using the “moran.test” function. A significance level of *P*-value  <  0.05 was used to assess the spatial autocorrelation. The main idea behind spatially autocorrelated data is that values are not independent of space. This concept is based on the first law of geography proposed by Waldo Tobler, according to which, “Everything is related to everything else, but near things are more related than distant things.”

$$ I=\frac{n}{s_o}\ \frac{\sum_{i=1}^n{\sum}_{j=1}^n{w}_{i,j}{z}_i{z}_j}{\sum_{i=1}^n{z}_i^2} $$

Where n is equal to the total number of features; S_o_ is the aggregate of all the spatial weights; z_i_ is the deviation of an attribute for feature I from its mean (x_i_-X); and w_i,j_ is the spatial weight between feature i and j. The Moran’s I score ranges from − 1 (dispersed) to 1 (clustered). A value of 0, or very close to 0, refers to random distributions. Positive autocorrelation suggests that points with similar attribute values are closely distributed in space, whereas negative spatial autocorrelation suggests that closely associated points are more dissimilar in spatial terms. By applying the Monte Carlo simulation computational technique, Moran’s I was permuted 999 times to determine the significance using multiplication.

The global spatial autocorrelation does not reveal the existence of regional spatial patterns. Therefore, to visualize spatial clustering, Local Indicators of Spatial Autocorrelation (LISA) maps were created using local Moran’s index calculations [29]. LISA statistic was calculated for each observation and cluster, with the significance level at *P*  <  0.05. The LISA statistic indicates the extent of significant spatial clustering of similar or dissimilar values around a spatial feature. It is provided by the following formula:

$$ {I}_i=\frac{n\left({x}_i-\overline{x}\right)\ {\sum}_{j=1}^n{w}_{ij}\left({x}_j-\overline{x}\right)}{\sum_{i=1}^n{\left({x}_i-\overline{x}\right)}^2} $$

The parameters for the LISA statistics are the same as those for Moran’s I. In fact, the sum of the LISA statistics for all spatial features is proportional to the global Moran’s I. A positive *I*_*i*_ value indicates spatial clustering of similar values around a spatial characteristic, whereas negative values indicate a clustering of dissimilar values around a spatial feature.

**Fig. 2:**
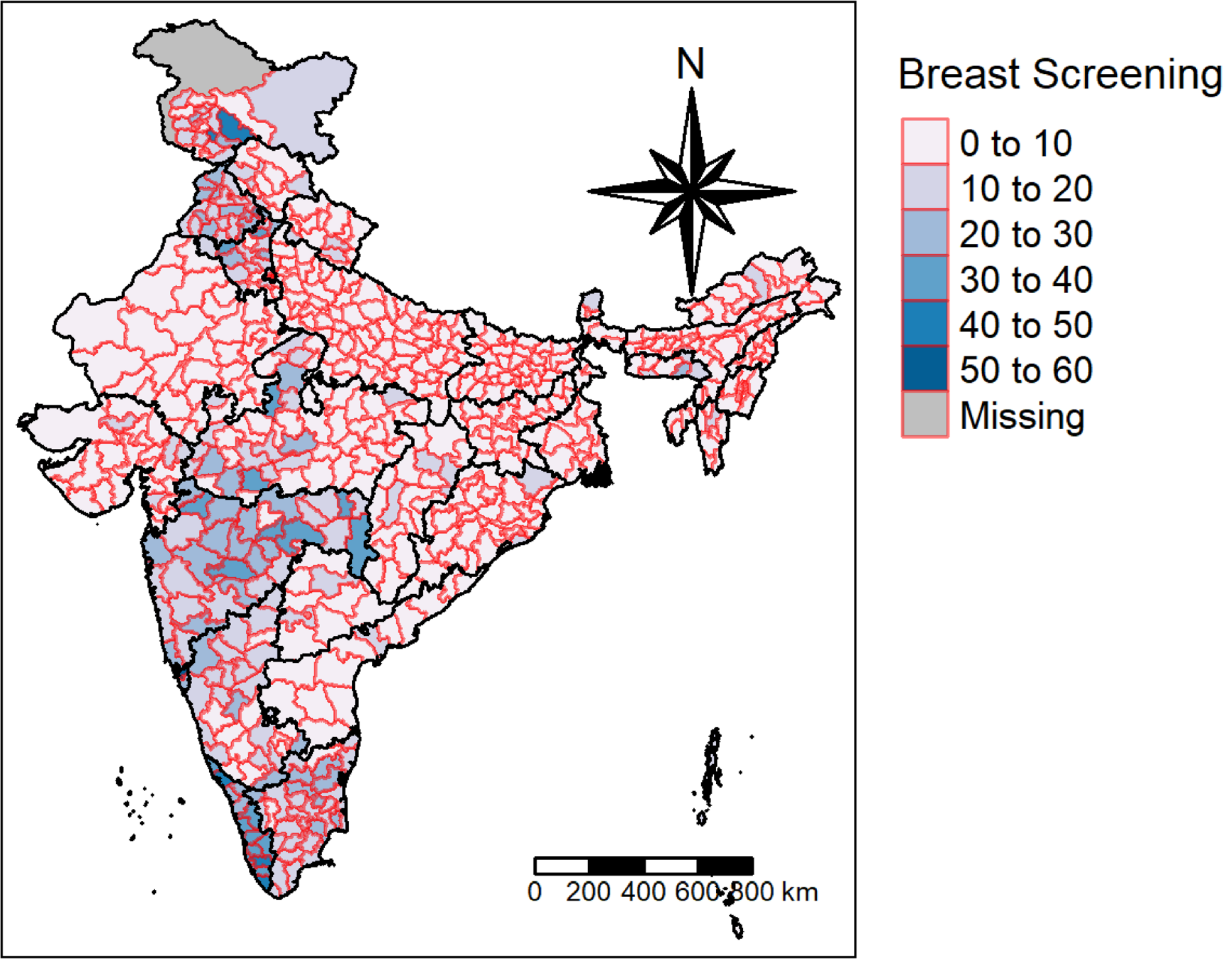
Prevalence of Breast Screening

**Fig. 3:**
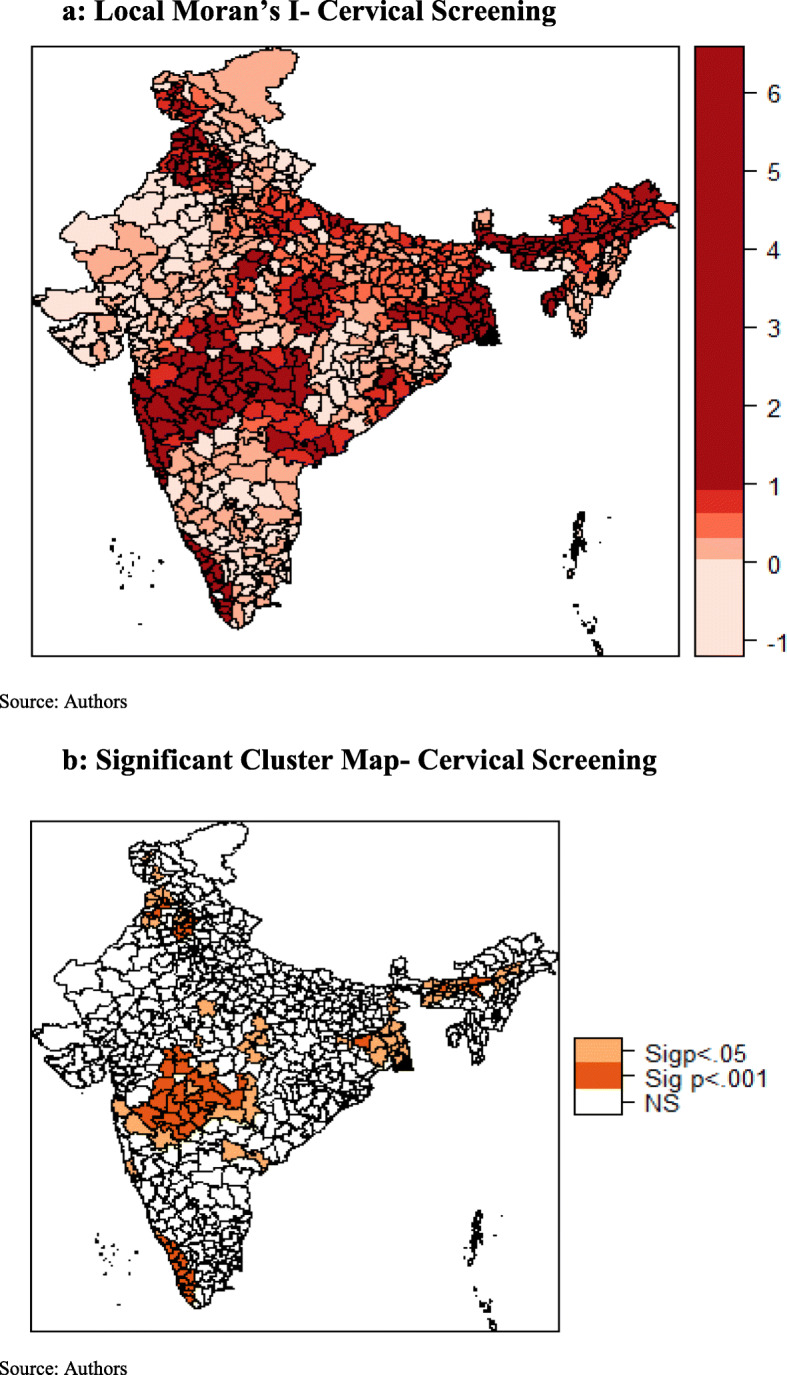
**a** Local Moran’s I- Cervical Screening. **b** Significant Cluster Map- Cervical Screening

Four types of spatial associations can be derived from this statistic and plotted in Moran’s scatter plot, where high-high (HH) and low-low (LL) types of spatial clustering denote similar values, and high-low (HL) and low-high (LH) types of spatial clustering indicate dissimilar values, referred as, spatial outliers [29] (see Fig. 2 & Fig. 3 in appendix).

Univariate Local Indicators of Spatial Association (LISA) measures the correlation of neighborhood values around a specific spatial location. It determines the extent of spatial non-stationarity and clustering present in the data. It is given by:

$$ {I}_i={z}_i\sum \limits_j{w}_{ij}{z}_j $$

Thus, the data set and the shapefiles were imported to R studio to calculate Moran’s I and generate detailed Local Indicators of Spatial Association (LISA) maps to study the spatial variations and conduct the spatial analysis. Then, the spatial error model was used to scrutinize for the presence of a spatial relationship, which shows that a value observed in one location depends on the values found at the nearby sites, indicating a spatial dependence. Spatial data may show spatial dependence in the variables and error terms. When spatial dependence is present in the error term, a spatial autoregressive specification for this dependence is typically assumed.

Spatial error model incorporates spatial effects through error term.

$$ \mathrm{Y}=\mathrm{x}\upbeta +\upvarepsilon $$

$$ \upvarepsilon \kern0.5em =\kern0.5em \uplambda\ \mathrm{W}\upvarepsilon +\kern0.5em \upxi $$

ɛ is the vector of error terms, spatially weighted using the weight matrix (W).

λ is the spatial error coefficient.

ξ is a vector of uncorrelated error terms.

If there is no spatial correlation between the errors, then λ = 0.

The spatial error model tells us only that there is an unexplained spatial structure to the residuals, not what caused them. It may offer better estimates of the model parameters and their statistical significance, but it does not presuppose any spatial process generating the patterns in the screening values. A different model that explicitly tests for whether the screening at a point is functionally dependent on the values of neighboring points is the spatially lagged model [30]. It is given by: *y* = ρ W y + Xβ + ɛ.

where **y** is the endogenous variable, X is a matrix of exogenous variables, and W is the spatial weights matrix.

## Results

### Geographical distribution of cervical and breast screening

Figures 1 and 2 display the prevalence of cervical and breast screening across the districts of India. According to the NFHS report, 22% of women have undergone a cervical examination, whereas the corresponding figure for breast examination was 10%. The pattern of cervical screening indicates that the southern region, Kerala particularly, has a major contribution followed by districts from Maharashtra. The distribution curve of the prevalence of cervical screening and the districts shows that the majority of the districts fall in the range of 10 to 20%. A majority of the districts in Kerala have a high uptake of breast cancer screening, whereas North Goa has the maximum share. The distribution curve exhibits that most of the districts fall in the range of 0 to 10%.

### Descriptive statistics

Table 1 presents the summary statistics of the variables used in the analysis. The average values of cervical screening and breast screening across the districts were 22 and 10% respectively. Little variation was seen in the average values of women having multiple partners, those having parity more than 3, and those consuming alcohol (SD = 5, 8, 6%). Hindu women and those who were literate, currently married, and residing in rural areas had mean proportion values above 70% across the districts of India. Descriptive statistics for all the characteristics under study at the individual level are provided in the appendix Table 1.

**Table 1:** Summary Statistics of Dependent and Independent Variables

***N*** = 640							
Characteristics	Mean	Median	Standard Deviation	Characteristics	Mean	Median	Standard Deviation
Cervical Screening	21.96	18.47	14.62	Rich	38.71	33.98	24.26
Breast Screening	9.66	6.71	8.47	Oral Contraception	14.13	9.87	12.26
Literate	72.39	73.85	14.18	Tobacco	7.13	3.66	10.78
Currently Married	71.96	72.78	5.54	Insurance	18.96	10.99	20.13
Hindu	74.58	85.33	27.69	Multiple Partners	4.20	2.81	4.75
General Caste	21.77	17.48	18.29	Parity> 3	13.14	12.37	7.58
Rural	71.57	77.98	21.66	Alcohol	2.59	0.28	6.43

Table 2 illustrates Moran’s I values for the dependent and independent variables incorporated in the study. Moran’s I value for cervical and breast screening were 0.61 and 0.55 respectively, indicating a high spatial autocorrelation across the districts of India. Moran’s I for the independent variables ranged between 0.81 (for districts with the percentage of tobacco consumption) and 0.41 (for districts with the percentage of women having multiple partners).

**Table 2:** Moran’s I for Dependent and Independent Variables

Characteristics	Moran’s I	Characteristics	Moran’s I
Cervical Screening	0.610	Rich	0.702
Breast Screening	0.555	Oral Contraception	0.765
Literacy	0.690	Tobacco	0.813
Currently Married	0.570	Insurance	0.735
Hindu	0.749	Alcohol	0.580
General caste	0.553	Parity > 3	0.753
Rural	0.418	Multiple Partners	0.413

Note: above values are significant at *p* value < 0.01

### Univariate LISA maps

**Fig. 4:**
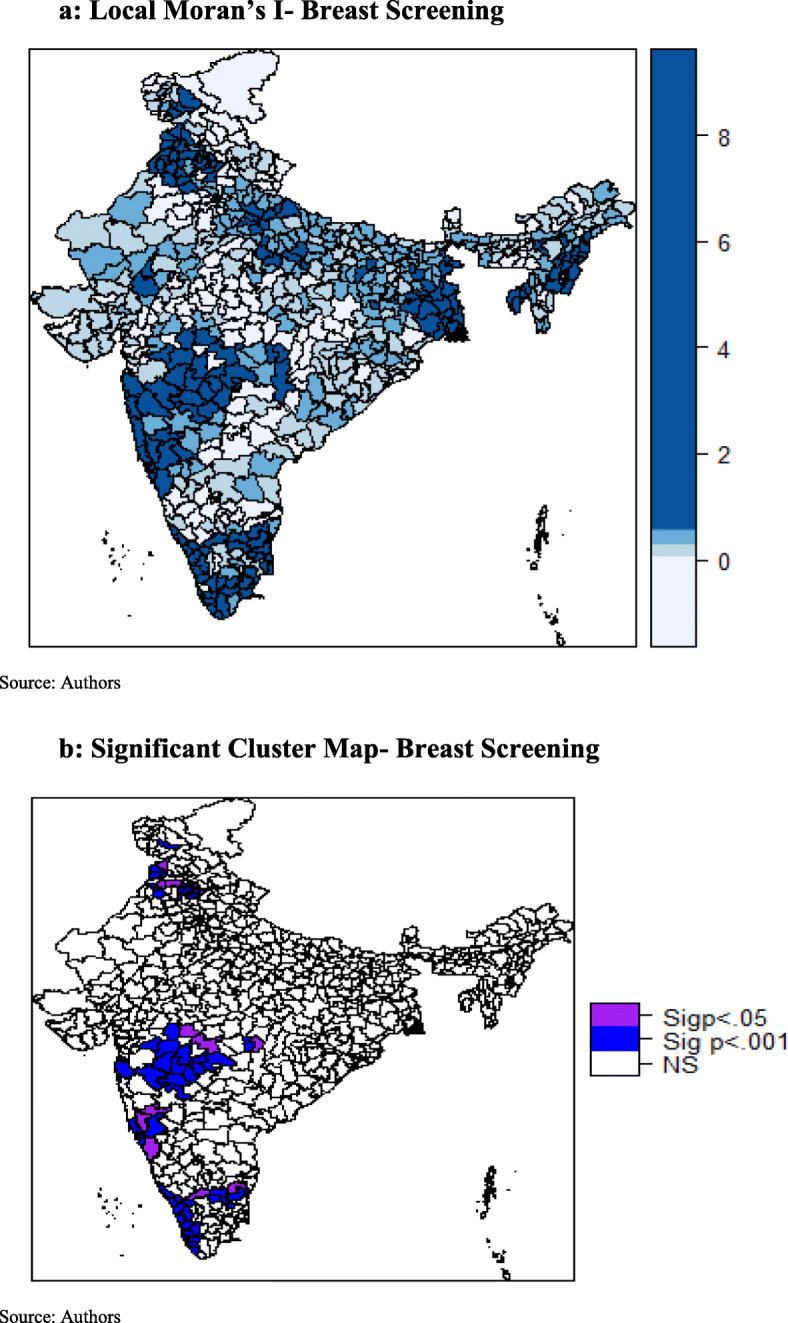
**a** Local Moran’s I- Breast Screening. **b** Significant Cluster Map- Breast Screening

Local Moran’s I value for cervical and breast screening were plotted in Fig. 3 a and Fig. 4 a respectively. Whereas, the significant local clusters of cervical and breast screening were presented in Fig. 3 b and Fig. 4 b respectively. A highly dense clustering of cervical screening could be seen in the districts of Kerala, Maharashtra, Assam, Punjab, Jammu and Kashmir, and West Bengal (Fig. 3b). A few clusters were also observed in the districts of Madhya Pradesh and Uttar Pradesh. As seen in Fig. 4b, clustering of breast screening could be found in the districts of Kerala, Tamil Nadu, Karnataka, Maharashtra, Punjab, and Jammu and Kashmir.

### Ordinary least squares and spatial regression models

Illustrated in Table 3, the OLS regression model identified that, higher socio-economic status was found to be significantly positively associated with cervical screening across the districts, as was having insurance. This was also the case among districts having a higher proportion of currently married women, belonged to a general caste, and resided in rural areas in the districts. In contrast, use of oral contraceptives, having multiple partners, having parity above three, and being a Hindu woman were seen to have a significant negative association with cervical screening in the districts.

**Table 3:** Factors influencing Cervical and Breast Screening (Ordinary Least Square Model)

Characteristics	OLS Cervical	*P* value	OLS Breast	*P* value
Literate	− 0.015	0.877	0.130	< 0.001
Currently Married	0.262	0.009	0.162	< 0.001
Hindu	− 0.096	< 0.001	− 0.054	< 0.001
General Caste	0.103	0.001	0.054	0.005
Rural	0.127	< 0.001	0.023	0.120
Rich	0.251	< 0.001	0.134	< 0.001
Oral Contraception	−0.571	< 0.001	− 0.245	< 0.001
Tobacco Consumption	− 0.006	0.881	− 0.145	< 0.001
Insurance	0.079	0.004	0.034	0.051
Multiple Partners	−0.394	< 0.001	–	–
Parity> 3	− 0.403	< 0.001	–	–
Alcohol	–	–	− 0.015	0.901
Obese	–	–	− 0.325	< 0.001
Model Estimates				
Multiple R Squared	0.389		0.342	
Adjusted R Squared	0.379		0.331	
AIC	4968		4314.4	

Table 3 shows the effect of female literacy on breast screening at the district level, which at 0.13 was found to be positively significant. This implies that the education of women and preventive healthcare-seeking behavior goes hand in hand. Districts with higher percentage of currently married women were 0.16 times more likely to go for breast examination. A similar positive association was observed among districts with a higher percentage of general caste women, women from economically prosperous districts, districts with higher insured women, and women from rural areas. Use of oral contraceptives (− 0.24) and consumption of tobacco (− 0.15) had a significant negative association with breast screening. Even adiposity and breast screening shared a negative association (− 0.32) with each other at the district level.

The variance inflation factor (VIF) estimates showed that there was very little multicollinearity among the independent variables (Table 2 Appendix). Moran’s I value of 0.42 (*p* < 0.01) and 0.34 (*p* < 0.01) for cervical and breast screening respectively indicated spatial autocorrelation of the residuals (Table 4). The Akaike Information Criterion (AIC) is a measure of the relative goodness of fit of a statistical model. For a set of models, the preferred model is the one with the minimum AIC value. The coefficient of determination (R-squared = 0.38 and 0.33 for cervical and breast screening respectively) indicated that the OLS model was not the best fit.

**Table 4:** Spatial Autocorrelation of Residuals for Cervical and Breast Screening

Residuals	Moran’s I	
	Cervical	Breast
OLS	0.422 ***	0.344 ***
Error	−0.039	−0.047

Note: *** = *p* < 0.01 ** = *p* < 0.05 * = *p* < 0.1

Table 5 shows that the Lagrange Multiplier (error) and the Lagrange Multiplier (lag) were significant and indicated the presence of a spatial dependence in the cervical and breast screening data. The robust error and the robust lag tests were also significant.

**Table 5:** Spatial Dependence for Cervical and Breast Screening

Test Statistics	Cervical	Breast
Lagrange Multiplier Error	293.65 ***	194.99 ***
Lagrange Multiplier Lag	266.96 ***	209.79 ***
Robust Lagrange Multiplier Error	37.28 ***	7.00 **
Robust Lagrange Multiplier Lag	10.59 **	21.79 ***
SARMA	304.24 ***	216.79 ***

Note: *** *p* < 0.001, ** *p* < 0.01

The spatial lag model provides Rho (ρ) as a coefficient parameter which measures the average effect on observations by their neighboring observations and thus reflects the spatial dependence inherent in the data. It was found to be statistically significant and had a positive effect for both the models (cervical and breast screening). As a result, the general model fit improved, as indicated by the higher values of log-likelihood. However, the significance of Breusch-Pagan test and the Likelihood Ratio test of spatial lag dependence for both the models revealed that even though the introduction of the spatial lag term led the model fit to improve, it could not eliminate the presence of spatial effects.

**Fig. 5:**
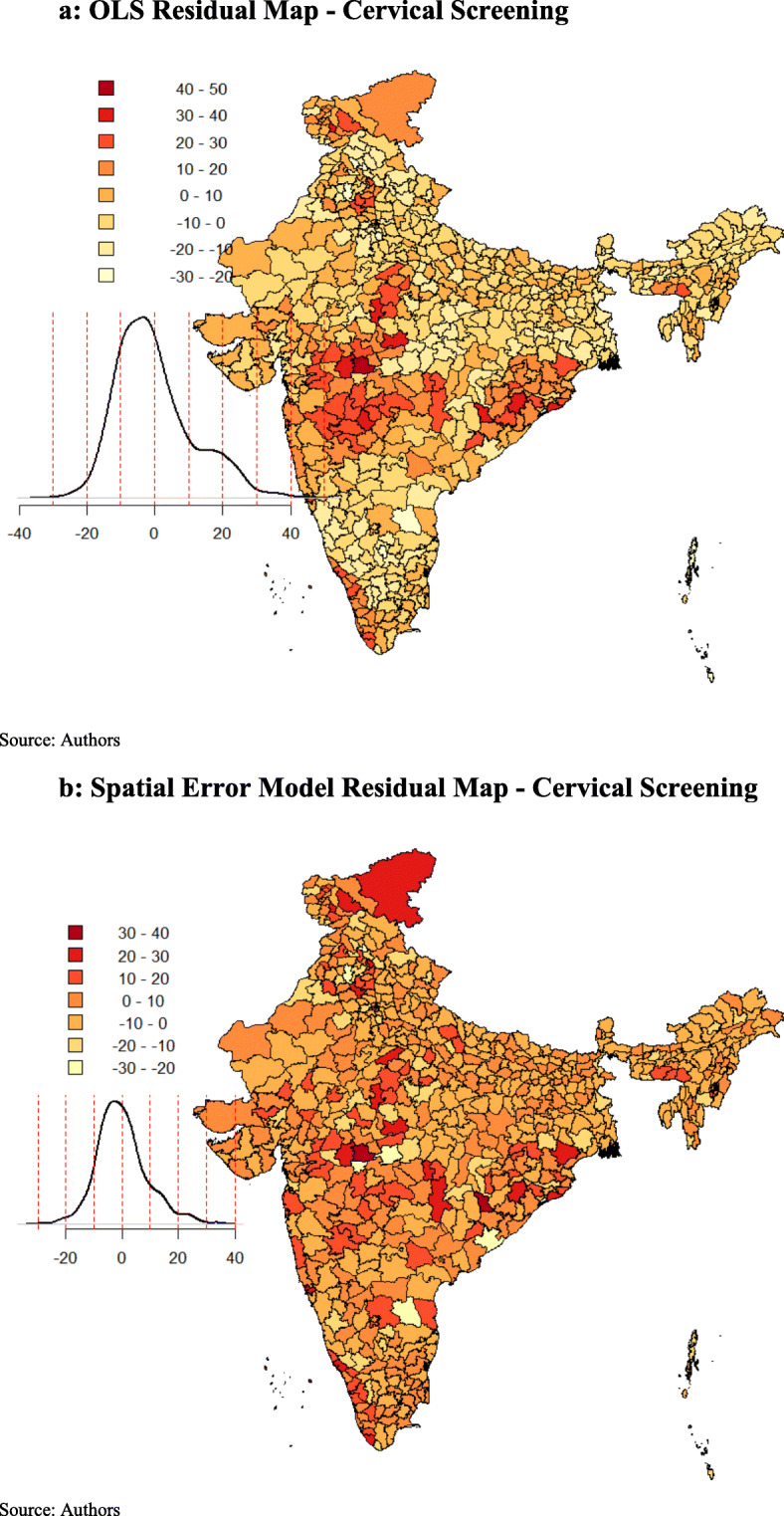
**a** OLS Residual Map - Cervical Screening. **b** Spatial Error Model Residual Map - Cervical Screening

**Fig. 6:**
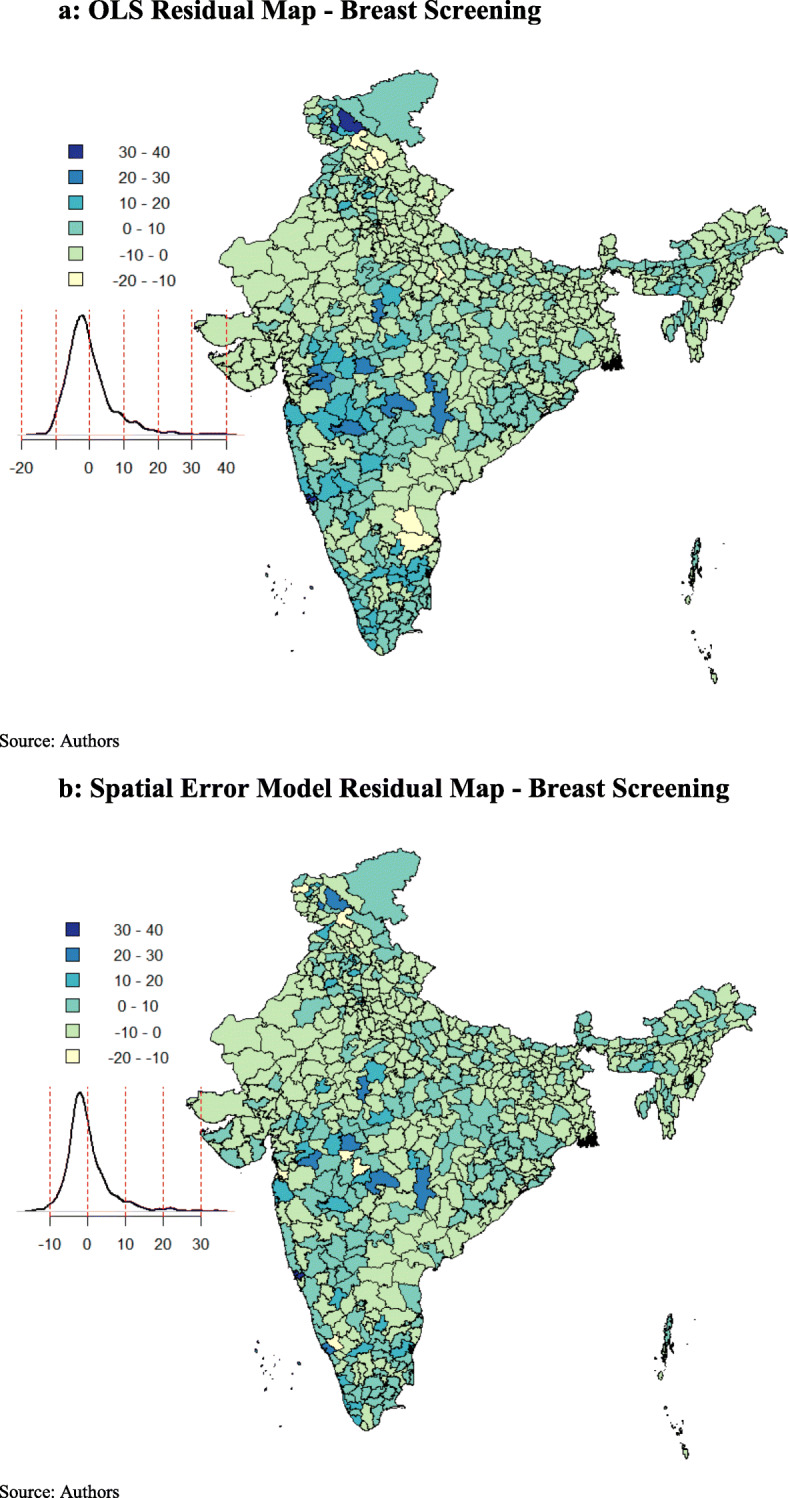
**a** OLS Residual Map - Breast Screening. **b** Spatial Error Model Residual Map - Breast Screening

The spatial error model provides λ (Lambda) as a coefficient on the spatially correlated errors. The model was highly significant, with a positive effect for both the models (cervical and breast screening). As a result, the model fit improved, as indicated by the higher values of log-likelihood. The Breusch-Pagan test and the Likelihood Ratio test of spatial error dependence were significant, indicating that the spatial effects in both the models (cervical and breast screening) were still present. However, both the spatial error and the spatial lag models were an improvement on the OLS model. The spatial error model appeared to fit the data better among all as the AIC score was lower and the log-likelihood value was greater for the spatial error model, employed for both cervical and breast screening. The residuals maps of OLS and spatial error model for cervical (Fig. 5a and b) and breast screening (Fig. 6a and b) indicated model improvement. The amount of clustering of the residuals reduced (the residuals appeared to be more randomly distributed), and the Moran’s I of the spatial error residuals was reduced from 0.42 to − 0.04 for cervical screening and from 0.34 to − 0.05 for breast screening. The maps indicated that the problem of spatial autocorrelation amongst the residuals was mainly solved by the spatial error model. Following this, we proceeded to the analysis, considering the coefficients of the spatial error model.

The results shown in Table 6 for the spatial error model demonstrate a statistically significant spatial autocorrelation (λ = 0.690) for cervical screening. Proportion of women having multiple partners (− 0.18) and using oral contraceptives (− 0.17) were negatively associated with women taking up cervical screening at the district level. The same was found to be the case among districts with the percentage of Hindu women. A significant positive association with cervical screening was found in districts where women were insured (0.09), were currently married, and districts with higher general caste female population. Women who resided in rural districts and those who belonged to higher economic classes also shared a positive association at the district level.

**Table 6:** Factors Influencing Cervical Screening (Spatial Error & Spatial Lag Model)

Characteristics cervical	Spatial Error	*P* value	Spatial Lag	*P* value
Literate	−0.068	0.550	0.009	0.943
Currently Married	0.302	0.001	0.214	0.018
Hindu	−0.083	0.003	−0.064	0.001
General Caste	0.083	0.006	0.073	0.004
Rural	0.156	< 0.001	0.087	0.001
Rich	0.332	< 0.001	0.159	< 0.001
Oral Contraception	−0.176	0.089	−0.266	< 0.001
Tobacco Consumption	−0.014	0.892	0.039	0.330
Multiple Partners	−0.182	0.062	−0.206	0.027
Parity> 3	− 0.057	0.967	−0.082	0.333
insurance	0.091	0.004	0.058	0.010
Model Estimates				
λ	0.690***		–	
ρ	–		0.590***	
LR Test Value	235.8 ***		215.12 ***	
Log-Likelihood	− 2353.1		− 2363.4	
AIC	4734.2		4754.8	
Studentized Breusch-Pagan	22.19**		29.42***	

Note: *** = *p* < 0.01 ** = *p* < 0.05 * = *p* < 0.1

In Table 7, the spatial error model employed for breast screening indicated a statistically significant spatial autocorrelation, with λ = 0.620. Districts with obese women were negatively associated (− 0.29) with the uptake of screening. A similar association was observed for districts with Hindu women, those who used oral contraceptives, and those who consumed tobacco. A significant positive association (0.12) with the uptake of breast screening was observed among districts with currently married women, those residing in rural areas, those belonging to a general caste, and those who were economically well-off.

**Table 7:** Factors influencing Breast Screening (Spatial Error & Spatial Lag Model)

Characteristics breast	Spatial Error	*P* value	Spatial Lag	*P* value
Literate	−0.011	0.767	0.056	0.014
Currently Married	0.116	0.007	0.117	0.010
Hindu	−0.038	0.007	−0.036	0.001
General Caste	0.064	0.001	0.046	0.005
Rural	0.062	0.001	0.019	0.217
Rich	0.179	< 0.001	0.085	< 0.001
Oral Contraception	−0.134	0.022	−0.137	0.001
Obese	−0.285	0.004	−0.222	0.003
Tobacco Consumption	−0.0732	0.050	−0.054	0.080
Alcohol	−0.005	0.932	−0.013	0.808
Insurance	−0.005	0.661	0.017	0.241
Model Estimates				
λ	0.620***		–	
ρ	–		0.534***	
LR Test Value	164.3 ***		161.5 ***	
Log-Likelihood	− 2062.04		− 2063.4	
AIC	4152.1		4154.9	
Studentized Breusch-Pagan	35.3***		30.52***	

Note: *** = *p* < 0.01 ** = *p* < 0.05 * = *p* < 0.1

The spatial autocorrelation (λ) came out to be statistically significant in the spatial error model, indicating that the relationship between screening and the independent variables at the macro-level (districts) may be misleading if spatial clustering is ignored. The spatial regression analysis enabled us to examine the spatial relationships of cervical and breast screening with their respective independent variables at the district level. It also helped us identify the determinants promoting the spatial pattern; in other words, factors that would help explain why and where screening was high.

## Discussion

The findings suggest that cancer screening behavior in the neighboring districts influence the screening rate in the observed districts as the coefficient of spatial error model (λ) was statistically significant. This can be justified by the communication between individuals in adjacent areas, who may tend to visit the same health services and thus having the similar behavior [31]. This study showed that screening was significantly associated with geographic location and there were observed differences in the patterns of spatial clusters of cervical and breast screening. In case of cervical screening, hotspots were majorly concentrated in districts of Kerala, Maharashtra, Assam, Punjab, Jammu and Kashmir and West Bengal. Few clusters were also observed in districts of Madhya Pradesh and Uttar Pradesh. For breast screening, districts of Kerala, Tamil Nadu, Karnataka, Maharashtra, Punjab, Himachal Pradesh, and Jammu and Kashmir exhibit spatial clusters. Contrary to spatial patterns of cervical screening, no hotspots were observed in districts of east and northeast region of India in case of breast screening.

Further, this study determined that at the district level; marital and economic status, area of residence and caste were common exposures spatially related to uptake of cervical and breast screening. One of the principal findings was the significant positive association between having insurance and undergoing cervical screening in both models. Health care coverage may affect the decision to undergo screening since those who are protected for such procedures pay less out of pocket than those whose costs are not adequately covered [32]. It’s worth noting that the marital status of women has a considerable role in influencing their decision of undergoing screening. For both models, cervical and breast screening showed a significant positive association among currently married females. Similar associations have been documented in other studies as well [15, 33]. Another crucial finding that emerged from our analysis was the statistically significant and positive association between high socioeconomic status and uptake of cervical and breast screening. This strongly resonates with the fact that the economic status of a woman profoundly influences her decision to undergo screening [15, 16]. For breast examination, this study points the negative area-based association between risky health behavior and screening. Districts with high level of tobacco consumption tend to have lower screening coverage. This finding corroborates to the fact that people involved in risky behaviors might have less consideration for their own health and thus give less preference for preventive health behavior like screening [34].

For cervical and breast screening, clear and distinct spatial clusters in districts of Kerala and Maharashtra, covering nearly the whole state, were hard to miss. This significant result may be attributed to the various steps taken by the Kerala state health department. For instance, the Kerala Police and the Swasthi Foundation, in association with the Aster Med-city, a leading quaternary care hospital in Kerala launched “Rakshaka Raksha,” a series of free camps to screen state police force for cancer and lifestyle diseases [35]. Kerala was also the first state in the Indian union to formulate a cancer control program along with the guidelines of the WHO as early as in 1988 (called 10-year action plan) [36]. Even the Panchayats in the state envisages cancer control activities as part of their People’s Plan Program. Thus, Kerala has turned out to be a role model for other states, with its focus on preventive health measures, in this case, screening. Similarly, in case of Maharashtra screening programs for cervical cancer are run by “Tata Memorial Hospital” since 1998 and “Prashanti Cancer Care Mission”, a non- profit organization provides care for breast cancer among females.

This study has some limitations, and future research should be encouraged in that direction. Firstly, NFHS-4 provides data for women in the reproductive age group of 15–49 years only. This prevented the study from analyzing women undergoing screening beyond this age group. Secondly, the scope of the paper limited us from providing any information as to whether women undergo screening of their own volition or due to external factors like government interventions. Thirdly, women opting for screening include both those who like to practice preventive behavior as well as those who are suffering from the disease itself. It is difficult to draw any inference due to data limitation.

## Conclusion

Even though cervical and breast cancer are preventable in nature through timely detection of precancerous lesions by using screening tests, there are geospatial variations in cervical and breast screening across districts of India. Thereby, this study highlights the importance of ensuring a region-specific and organ-specific approach towards control and prevention of cancer. Further, through thematic maps generation, the study indicates that geography must be considered while assessing disparities and allocating resources. This is in line with the current Indian government priorities, which as part of National Health Mission, for the first time have launched population-based prevention, screening, and control programs for cancers of the cervix and the breast. Also, determinants that contribute to the utilization of cervical and breast screening among women in India include being currently married, rural residence, belonging to general caste and high economic status. Being insured was positively associated with the uptake of cervical screening only. Effective implementation of population-based screening programs is the need of the hour and could be a way of improving the health outcomes of women in India.

## Supplementary information

**Additional file 1.** Fig. 1 Neighbor Weight Matrix Map. Figure 2 Moran scatter plot of cervical screening in distrcits of India. Figure 3 Moran scatter plot for breast screening in districts of India. Table 1: Socio – demographic characteristics of women. Table 2: Variance Inflation Factor (VIF) for Cervical and Breast Screening (Ordinary Least Square Model).

## Data Availability

Data and materials are freely available upon making an official request to DHS Team through the DHS website at https://dhsprogram.com/what-we-do/survey-Types/dHs.cfm

## Abbreviations

LISA: Local Indicators of Spatial Autocorrelation
OLS: Ordinary Least Square Regression
VIF: Variance Inflation Factor
AIC: Akaike Information Criterion
NFHS: National Family Health Survey
PPS: Probability proportional to Size
HPV: Human papillomavirus
