# Supplementary material for: An epidemiological study of cervical and breast screening in India: district-level analysis
Source: BMC Womens Health. 2020 Oct 7;20:225. doi: 10.1186/s12905-020-01083-6 (PMC7542863; doi:10.1186/s12905-020-01083-6)
Supplement: Supplementary file 1 — Additional file 1. Fig. 1 Neighbor Weight Matrix Map. Figure 2 Moran scatter plot of cervical screening in distrcits of India. Figure 3 Moran scatter plot for breast screening in districts of India. Table 1: Socio – demographic characteristics of women. Table 2: Variance Inflation Factor (VIF) for Cervical and Breast Screening (Ordinary Least Square Model). [file 12905_2020_1083_MOESM1_ESM.docx]

**Appendix**

**Fig 1**

**Neighbor Weight Matrix Map**


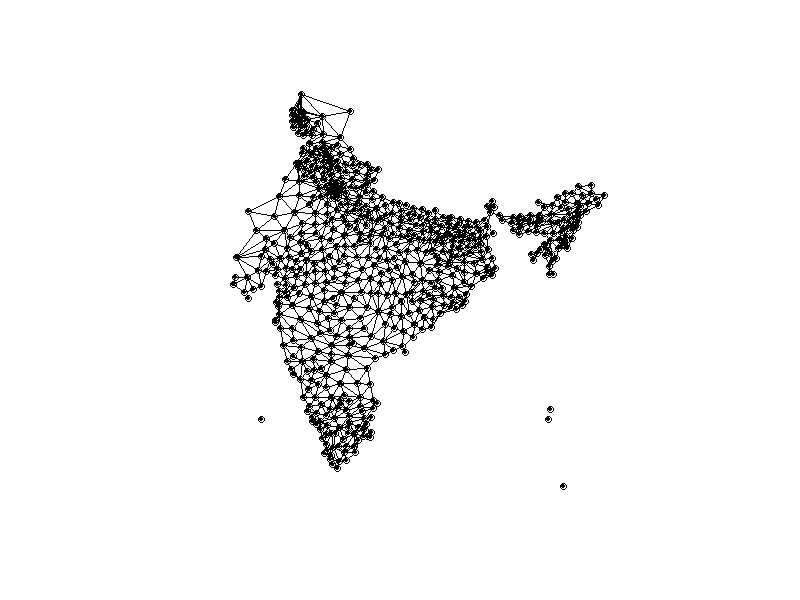


Source: Authors

Fig.1

Queen’s style contiguity neighbour weight matrix map. Neighbors are defined as the districts that share either a common border or a vertex with a given district (xi).

**Fig 2**

**Moran scatter plot of cervical screening in distrcits of India**


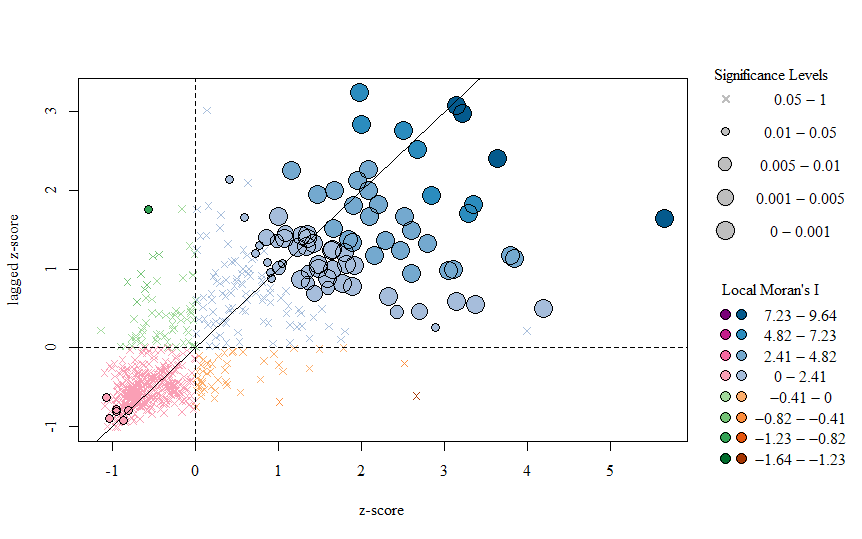


Source: Authors

**Fig. 3 Moran scatter plot for breast screening in districts of India.**

**
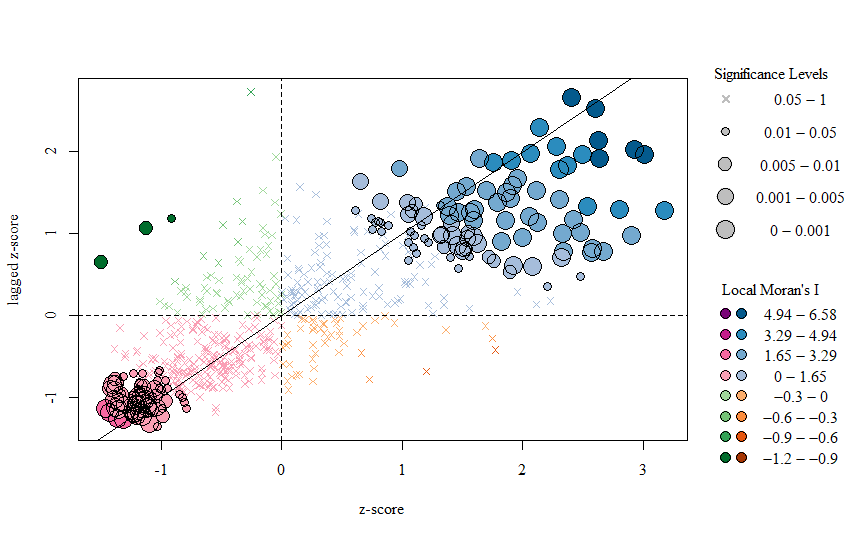
**

Source: Authors

**Table 1: Socio – demographic characteristics of women N=699,686**

| **Characteristics Cervical Screening** | | | | | **Characteristics Breast Screening** | | | | |
| --- | --- | --- | --- | --- | --- | --- | --- | --- | --- |
| Age Category | No |  | Yes |  | Age Category | No |  | Yes |  |
| 15-20 |  | 94.8 |  | 5.2 | 15-20 |  | 97.19 |  | 2.81 |
| 21-30 |  | 77.16 |  | 22.84 | 21-30 |  | 90.15 |  | 9.85 |
| 31-40 |  | 70.42 |  | 29.58 | 31-40 |  | 87.19 |  | 12.81 |
| 41-49 |  | 69.07 |  | 30.93 | 41-49 |  | 86.55 |  | 13.45 |
| Education |  |  |  |  | Education |  |  |  |  |
| Illiterate |  | 77.42 |  | 22.58 | Illiterate |  | 91.34 |  | 8.66 |
| Literate |  | 77.78 |  | 22.22 | Literate |  | 89.80 |  | 10.2 |
| Marital Status |  |  |  |  | Marital Status |  |  |  |  |
| Never Married |  | 97.64 |  | 2.36 | Never |  | 98 |  | 2 |
| Married |  | 71.62 |  | 28.38 | Married |  | 87.87 |  | 12.13 |
| Widow/Divorced |  | 75.18 |  | 24.82 | Widow/Divorced |  | 89.08 |  | 10.92 |
| Religion |  |  |  |  | Religion |  |  |  |  |
| Hindu |  | 77.73 |  | 22.27 | Hindu |  | 90.4 |  | 9.6 |
| Muslim |  | 81.09 |  | 18.91 | Muslim |  | 91.43 |  | 8.57 |
| Others |  | 68.7 |  | 31.3 | Others |  | 84.69 |  | 15.31 |
| Caste |  |  |  |  | Caste |  |  |  |  |
| SC |  | 78.95 |  | 21.05 | SC |  | 90.82 |  | 9.18 |
| ST |  | 79.71 |  | 20.29 | ST |  | 91.44 |  | 8.56 |
| OBC |  | 77.23 |  | 22.77 | OBC |  | 90.18 |  | 9.82 |
| others |  | 75.52 |  | 24.48 | others |  | 89.1 |  | 10.9 |
| Place of Residence |  |  |  |  | Place of Residence |  |  |  |  |
| Urban |  | 74.7 |  | 25.3 | Urban |  | 88.3 |  | 11.7 |
| Rural |  | 79.26 |  | 20.74 | Rural |  | 91.24 |  | 8.76 |
| Wealth |  |  |  |  | Wealth |  |  |  |  |
| Poor |  | 84.45 |  | 15.55 | Poor |  | 93.63 |  | 6.37 |
| Medium |  | 77.23 |  | 22.77 | Medium |  | 90.21 |  | 9.79 |
| Rich |  | 71.92 |  | 28.08 | Rich |  | 87.21 |  | 12.79 |
| Oral Contraception |  |  |  |  | Tobacco |  |  |  |  |
| Not Using |  | 83.5 |  | 16.5 | No |  | 90.17 |  | 9.83 |
| Oral |  | 82.54 |  | 17.46 | Yes |  | 91.07 |  | 8.93 |
| others |  | 68.18 |  | 31.82 | Insurance |  |  |  |  |
| Tobacco |  |  |  |  | No |  | 90.62 |  | 9.38 |
| No |  | 77.65 |  | 22.35 | Yes |  | 88.68 |  | 11.32 |
| Yes |  | 78.32 |  | 21.68 | BMI |  |  |  |  |
| Insurance |  |  |  |  | Normal and overweight |  | 90.48 |  | 9.52 |
| No |  | 78.83 |  | 21.17 | obese |  | 86.95 |  | 13.05 |
| Yes |  | 73.22 |  | 26.78 | Alcohol |  |  |  |  |
|  | | | | | No |  | 90.22 |  | 9.78 |
|  |  |  |  |  | Yes |  | 90.32 |  | 9.68 |

**Table 2: Variance Inflation Factor (VIF) for Cervical and Breast Screening (Ordinary Least Square Model)**

| Characteristics | VIF Cervical | VIF Breast |
| --- | --- | --- |
| Literate | 2.55 | 2.12 |
| Currently Married | 1.53 | 1.51 |
| Hindu | 1.78 | 1.57 |
| General Caste | 1.47 | 1.52 |
| Rural | 2.20 | 2.55 |
| Rich | 4.21 | 3.81 |
| Oral Contraception | 1.54 | 1.44 |
| Tobacco Consumption | 1.59 | 1.62 |
| Insurance | 1.51 | 1.32 |
| Multiple Partners | 1.32 | - |
| Parity> 3 | 2.93 | - |
| Alcohol | - | 1.28 |
| Obese | - | 2.03 |

A variance inflation factor indicates multicollinearity in ordinary least square regression. Multicollinearity is when there is correlation between independent variables. The VIF estimates shows that how much the variance of regression coefficient is inflated due to the presence of multicollinearity in the model. VIF value ranges from 1 and above. 1 denotes no correlation and values between 1-5 denotes moderate correlation among independent variables in the model.
